# Visual Scan Paths and Recognition of Facial Identity in Autism Spectrum Disorder and Typical Development

**DOI:** 10.1371/journal.pone.0037681

**Published:** 2012-05-29

**Authors:** C. Ellie Wilson, Romina Palermo, Jon Brock

**Affiliations:** 1 Macquarie Centre for Cognitive Science, Macquarie University, Sydney, New South Wales, Australia; 2 Department of Forensic and Neurodevelopmental Sciences, Institute of Psychiatry, King’s College London, London, United Kingdom; 3 School of Psychology, University of Western Australia, Western Australia, Australia; 4 Australian Research Council Centre of Excellence in Cognition and Its Disorders, Sydney, New South Wales, Australia; Monash University, Australia

## Abstract

**Background:**

Previous research suggests that many individuals with autism spectrum disorder (ASD) have impaired facial identity recognition, and also exhibit abnormal visual scanning of faces. Here, two hypotheses accounting for an association between these observations were tested: i) better facial identity recognition is associated with increased gaze time on the Eye region; ii) better facial identity recognition is associated with increased eye-movements around the face.

**Methodology and Principal Findings:**

Eye-movements of 11 children with ASD and 11 age-matched typically developing (TD) controls were recorded whilst they viewed a series of faces, and then completed a two alternative forced-choice recognition memory test for the faces. Scores on the memory task were standardized according to age. In both groups, there was no evidence of an association between the proportion of time spent looking at the Eye region of faces and age-standardized recognition performance, thus the first hypothesis was rejected. However, the ‘Dynamic Scanning Index’ – which was incremented each time the participant saccaded into and out of one of the core-feature interest areas – was strongly associated with age-standardized face recognition scores in both groups, even after controlling for various other potential predictors of performance.

**Conclusions and Significance:**

In support of the second hypothesis, results suggested that increased saccading between core-features was associated with more accurate face recognition ability, both in typical development and ASD. Causal directions of this relationship remain undetermined.

## Introduction

Autism spectrum disorder (ASD) is defined and diagnosed in terms of qualitative social and communicative impairments co-occurring with repetitive behaviours or restricted interests [1]. An ongoing debate is whether, in addition to this core triad of impairments, individuals with ASD also experience prosopagnosia – difficulty in perceiving and remembering facial identity (see [2] for a comprehensive review). Numerous studies have shown that identity recognition is impaired in groups of individuals with ASD compared with non-ASD comparison groups [3]–[10]. However, there have been a similar number of studies reporting non-significant group differences in face recognition performance [11]–[13], leading some researchers to argue that, in fact, face recognition in ASD is *unimpaired* [14]. Recently, a number of studies have suggested that there is in fact considerable heterogeneity within the autism population and that, while some individuals may have severely impaired face recognition, others are functioning well within the normal range, at least on laboratory tasks [15]–[19].

In the current study, we sought to understand the nature of individual differences in facial identity recognition in individuals with and without autism. Specifically, we investigated the association between face recognition performance and the patterns of eye-movements made by participants during the encoding of face stimuli. In a pioneering eye-tracking study, Yarbus [20] showed that adults typically scan faces in a highly stereotyped fashion, fixating on the core features (eyes, nose, and mouth), with a particular bias towards the eye region. Subsequent research has shown similar scan paths when infants view faces [21]. More recently, *abnormal* face scanning has been associated with prosopagnosia, a condition characterized by impaired facial identity recognition [22]–[24]. A number of authors have also posited a link between abnormal face scanning in ASD and deficits in facial identity recognition [7], [25]. Whilst there is evidence that groups of individuals with ASD exhibit atypical visual scanning, empirical evidence for an association at the individual level is lacking.

Here, we specifically investigate two plausible hypotheses linking face recognition impairments to aberrant scan paths in ASD. The first hypothesis is that poor recognition of facial identity is a function of a reduced tendency to fixate on the eye region of faces. The eyes are considered to be one of the most important features for recognizing identity, as well as other face attributes such as emotion, age and gender [26]–[29]. In line with this, a number of eye-tracking studies have reported that, when viewing faces, children and adults with ASD spend less time looking at the eyes and more time looking at the mouth than typically developing individuals [30]–[36], although some studies have not replicated this [37]–[40]. Here, we predicted that an increase in looking at the eye-region would correlate with better facial identity recognition ability.

Charawarska and Shic [25] directly investigated this link in toddlers with ASD, but the results were in the opposite direction to predictions. A greater bias towards fixating the eyes was associated with poorer facial identity recognition, as assessed with a preferential looking paradigm, Notably, in contrast to studies of older children and adults discussed above, the ASD toddlers spent *more* time focusing on the eyes at the expense of the mouth compared to typically developing controls. Thus, it is important to investigate this association in an older sample of children.

Whilst typical individuals tend to fixate more on the eyes than other facial features, they nevertheless distribute their visual attention between the core features of the face (i.e. eyes, nose, and mouth) [41]–[43]. Saccading between facial features is thought to generate a unified percept of features and their configuration [44], [45]. Our second hypothesis, therefore, is that the face recognition difficulties seen in some individuals with ASD might be a function of inappropriate distribution of attention across the core facial features. Direct evidence here is scarce. In a study with five ASD adults, Pelphrey et al [46] reported disorganized, erratic and undirected scanning strategies, with fewer fixations on the core features, and more on other areas of the face (forehead, cheeks, chin). In addition, the ASD participants were impaired at recognizing facial emotion, leading the authors to hypothesize that aberrant face scanning and poor emotion processing were related. However, the small sample size in this study prevented analysis of associations at the level of individuals. Furthermore, the link between scanning and *identity* recognition was not considered.

To test these two competing hypotheses, we recorded the eye movements of children with ASD and typically developing children while they viewed photographs of unfamiliar faces. We then tested participants’ recognition memory for these faces and determined whether measures of eye gaze or dynamic scanning between facial features were able to predict within-group individual variation in performance.

## Methods

### Ethics Statement

All participants provided written informed consent to take part in this study. The research was approved by the Macquarie University human ethics committee.

### Participants

Eleven participants (7 male) were recruited through Autism Spectrum Australia (ASPECT) and Macquarie University Special Education Centre (MUSEC). All eleven children met criteria for ASD according to the DSM IV [1], and each child achieved scores indicative of an ASD on the Social Communication Questionnaire (lifetime version; SCQ) [47]. Eight participants had been previously diagnosed using the Autism Diagnostic Interview Revised (ADI-R) [48] or the Autism Diagnostic Observation Schedule (ADOS) [49]. A diagnosis of ASD was conferred in the remaining three children with the Childhood Autism Rating Scale (CARS) [50]. Six children were classified as autistic, and the remaining five with Asperger syndrome. Two additional participants that were tested did not follow instructions and were excluded from all analyses. A third participant was excluded because inspection of his eye-tracking data revealed that he only spent 53% of the time looking at the screen, and he performed at chance level on the recognition memory task.

Previous studies, both of ASD and typical development, indicate that performance on face recognition tasks is strongly associated with chronological age, but is relatively independent of IQ [18], [51]–[54]. The ASD participants were matched to a group of eleven typically developing (TD) children (6 male) for chronological age.

Receptive grammar skills were assessed using the Test for Reception Of Grammar (TROG-2) [55]. Children were also administered the matrices subscale of the Weschler Scale of Intelligence (WASI matrices) [56], which measures nonverbal fluid reasoning and general intellectual ability. This test is comparable to the widely used Raven’s matrices but has the advantage of extensive normative data. Participant characteristics are provided in Table 1.

**Table 1:** Participant characteristics.

Variable. Test Mean (SD)	ASD (N = 11)	TD (N = 11)	t-value. *p* (df = 20)
Age (years)	10.21 (2.00) 7.58–14.47	10.54 (2.04) 7.75–15.00	0.39 (*p* = 0.76)
TROG 100 (15)	82.55 (20.09) 55–109	106.55 (12.53) 81–123	3.36 (*p*<0.01)
WASI matrices 50 (15)	45.72 (11.60) 25–64	57.27 (7.95) 39–72	2.72 (*p* = 0.01)
SCQ (lifetime version)	22.27 (4.73) 15–32	3.27 (1.48) 1–5	−12.60 (*p*<0.001)

Means, standard deviations, and ranges of scores. t-values indicate the difference between ASD and TD scores.

### Design

The experiment consisted of four old-new recognition tests. On each test a set of 20 faces were learnt during an *Encoding phase* and recognition was assessed in a subsequent *Recognition phase* in which each of the old faces was paired with a similar distractor. A two alternative forced-choice (AFC) design was used to eliminate biases in responding “old” that may vary between ASD and TD children. At the end of each old-new test the participants viewed five photographs of social scenes for 10 seconds each; data from this component is reported elsewhere [17].

### Stimuli

Greyscale photographs of one hundred and sixty (80 female) Caucasian individuals aged between eighteen and forty were selected from the Glasgow Face Group database (www.psy.gla.ac.uk/~mike/facerec.html). Whilst there is evidence for an own-age bias in recognition, with TD children more accurate at recognising faces close (∼2 years) to their own age (e.g. [57]), we assume that scan paths on faces of different ages would be similar, although no studies have directly investigated this. Photographs were selected to form pairs matched on sex, hairstyle, mouth position (i.e. open or closed; smiling or not), and eye gaze direction, as well as previously collected ratings of distinctiveness. The faces were presented in oval windows that were 10 cm×8 cm in size, so that each face was approximately 9×11 degrees of visual angle, when viewed from a distance of approximately 50 cm. One member of each pair was designated the target and the other was the distracter.

In 50% of photographs the person was looking directly at the camera (Direct gaze) while in the other half the person was looking to the side (25% left, 25% right) but still facing the camera (Averted gaze). We were originally interested to test whether children with ASD showed the typical advantage for recognition of faces with direct gaze [58], [59]. However, in the event, the TD group did not show this expected effect, so we ignored this manipulation in all further analyses.

### Procedure

Eye movements were recorded with a remote Eyelink 1000 (SR-Research) remote eye-tracking camera, which was placed under a flatscreen monitor and recorded the point of gaze of the right eye at a sampling rate of 500 Hz. Participants wore a small sticker on their forehead which enabled the eye tracker to continually monitor their head position. A nine-point calibration method was used to calibrate and validate eye-tracking for each participant.

The experiment was conducted in four blocks with calibration completed at the beginning of each. Each block included an encoding phase (20 target faces) followed by a recognition phase (20 target-distracter pairs). Three practice trials were completed prior to the first block to ensure participants could complete the task. The experiment took between 30 and 60 minutes to complete.

#### Encoding phase

**Figure 1:**
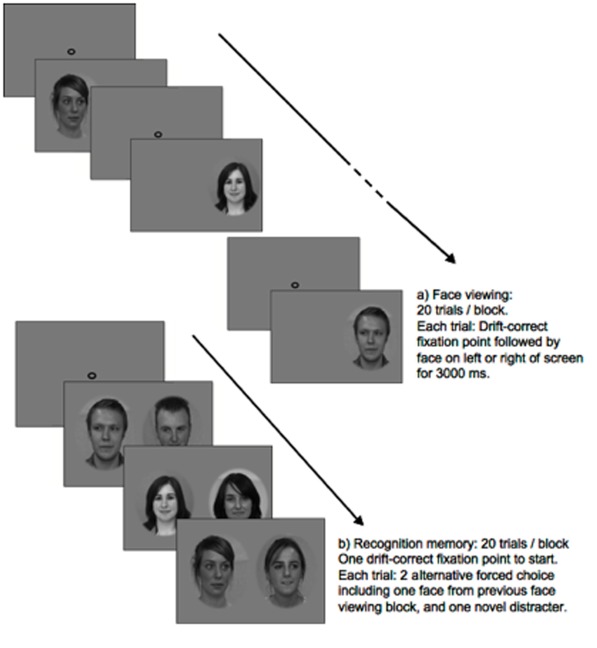
Trial sequence for recognition memory test (direct eye gaze condition). (a) Face viewing, 20 trials/block; (b) 2-alternative forced-choice recognition memory test, 20 trials/block.

Each trial started with a drift correction, which required participants to fixate on a centrally positioned spot. Next, a face was shown either to the left or right of the central fixation spot, in a pseudo-random order. Faces were positioned so that the bottom of the nose was horizontally in line with the central fixation point (Figure 1a). By fixating off the face to begin with, participants were not forced to look at any region of the face. Participants were instructed to look at each face and try to remember it. Each face was presented for 3 seconds, which is in the middle of the range of presentation durations of previous tests of unfamiliar face recognition (e.g., [24], [44]). Pilot testing indicated that this exposure duration would minimize the chances of participants performing at either ceiling or floor levels.

#### Recognition memory test

This was a two AFC recognition memory test (Figure 1b). On each trial a pair of faces was presented, one of which was a target face from the preceding encoding phase, the other being the paired distracter. Participants indicated whether they recognized the face on the left or right by pressing the ‘Z’ or ‘/’ key, which were marked with coloured stickers. Accuracy rather than speed was encouraged, and the face pairs remained on screen until the participant responded. Trials were presented in a fixed pseudo-random order that was different to the order at encoding, and the position of the target was fully counterbalanced.

### Analysis

#### Age-standardized face identity recognition scores

In the absence of normative data for the face recognition task, we constructed age-standardized scores via a linear regression analysis, combining data from the 11 TD children with data from a further 20 TD children (8 male, mean age  = 9.03, SD  = 2.01) who completed the same task but without the eye-tracking component (procedures were identical apart from the absence of the calibration and drift correct routines). For each participant in the experiment, we calculated an age-standardized score by subtracting the predicted scores, based on the regression equation of the combined TD group (N = 31), from their actual scores and then dividing the residual by the standard error of the estimate (see [60] for details of implementation).

#### Eye-movements

Eyelink Data Viewer software was used to analyse eye-movements to faces in the *Encoding* phase only (similarly to [61]). In the *Recognition phase*, eye-movement analysis was complicated by the fact that two faces were present on the screen and the variable interval between presentation and response.

For each face, the following interest areas were coded: Eyes (below the eye brow); Mouth; Nose; Non-features (remainder of the face); Hair region (including ears if visible); background. An ellipse was used for the face region and all other interest areas were individually hand-drawn. The Eye region was divided into left and right eyes by distinguishing between fixation points that fell to the left or right of a central vertical line.

The ‘gaze time’ for each interest area was calculated by calculating the time spent looking at each interest area across the whole experiment and then calculating the average gaze time per trial.

To quantify the amount of saccading between features, we devised a Dynamic Scanning Index, which was incremented each time the participant saccaded into and out of one of the four core-feature interest areas (left-eye, right-eye, nose, and mouth). Saccades were identified if instantaneous velocity exceeded 30 deg/sec, or if acceleration exceeded 8000 deg/sec^2^. For example, the saccade sequence: ‘Central Fixation’ to ‘Left Eye’ to ‘Hair’ to ‘Mouth’ to ‘Left Eye’ to ‘Rest of Face’, would score a Dynamic Scanning Index of 3 because there were 3 instances of a core interest area being saccaded into and out of. Multiple fixations on the same feature would not increment the Dynamic Scanning Index unless participants saccaded to other regions in between. For each subject, we calculated the total Dynamic Scanning Index across the whole experiment and then calculated the average Dynamic Scanning Index per trial; from here on, ‘Dynamic Scanning Index’ refers to the average score per trial.

## Results

Due to a technical error, one ASD participant’s experiment was terminated after 64 of the trials. The analyses report percent or average scores, therefore this participant’s scores were calculated according to the number of trials they completed, and they are included in all final analyses.

### Performance on the Recognition Memory Test

**Figure 2:**
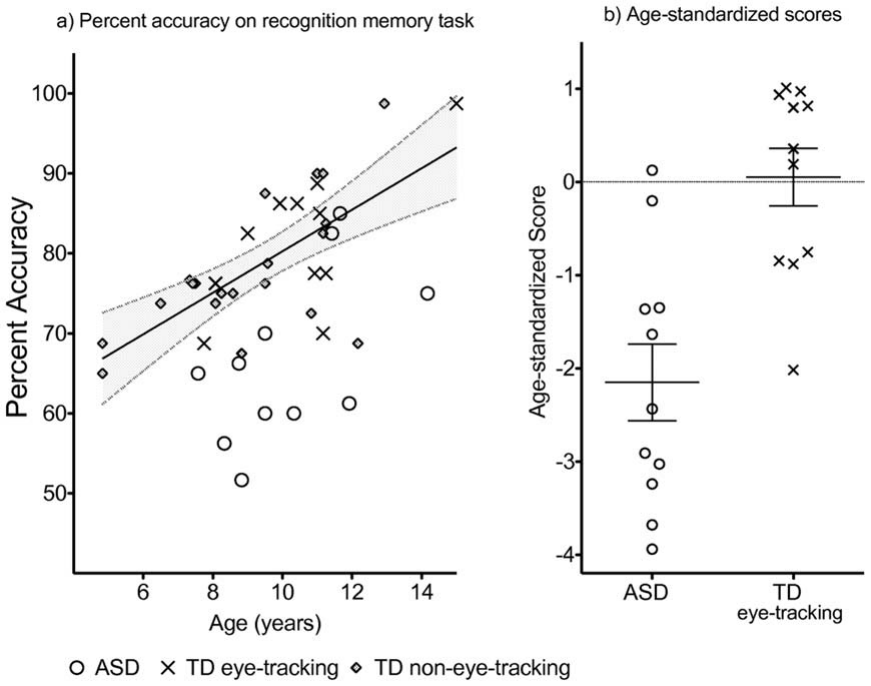
Scores on the recognition memory task. a) Percent accuracy and age of participants in the ASD, TD eye-tracking and TD non-eye-tracking groups. Regression line is based on all the TD participants’ scores (R^2^ = 0.45). b) Age-standardized scores of the ASD and TD eye-tracking groups. Bars show group means and standard error means.

Figure 2 shows the raw scores of all participants plotted as a function of age, as well as the derived age-standardized scores. For the TD group, standardized scores were close to zero. For the ASD group, standardized scores were significantly below zero, t (11)  =  −5.22, *p*<0.001. Correlational analyses showed that, as expected, standardized scores were uncorrelated with standardized WASI matrices or TROG scores, or with SCQ scores.

One ASD participant performed at chance level on the recognition task. Review of his eye-tracking data confirmed that he was looking at the screen 81% of the time during the encoding phase, providing confidence that he was attending to the task. All the analyses that examined relationships between eye-movement behaviour and recognition performance were re-calculated excluding this participant. All significant results remained and therefore his data was included in the reported results.

### Visual Scanning of Faces During Encoding

#### Looking time on interest areas

**Figure 3:**
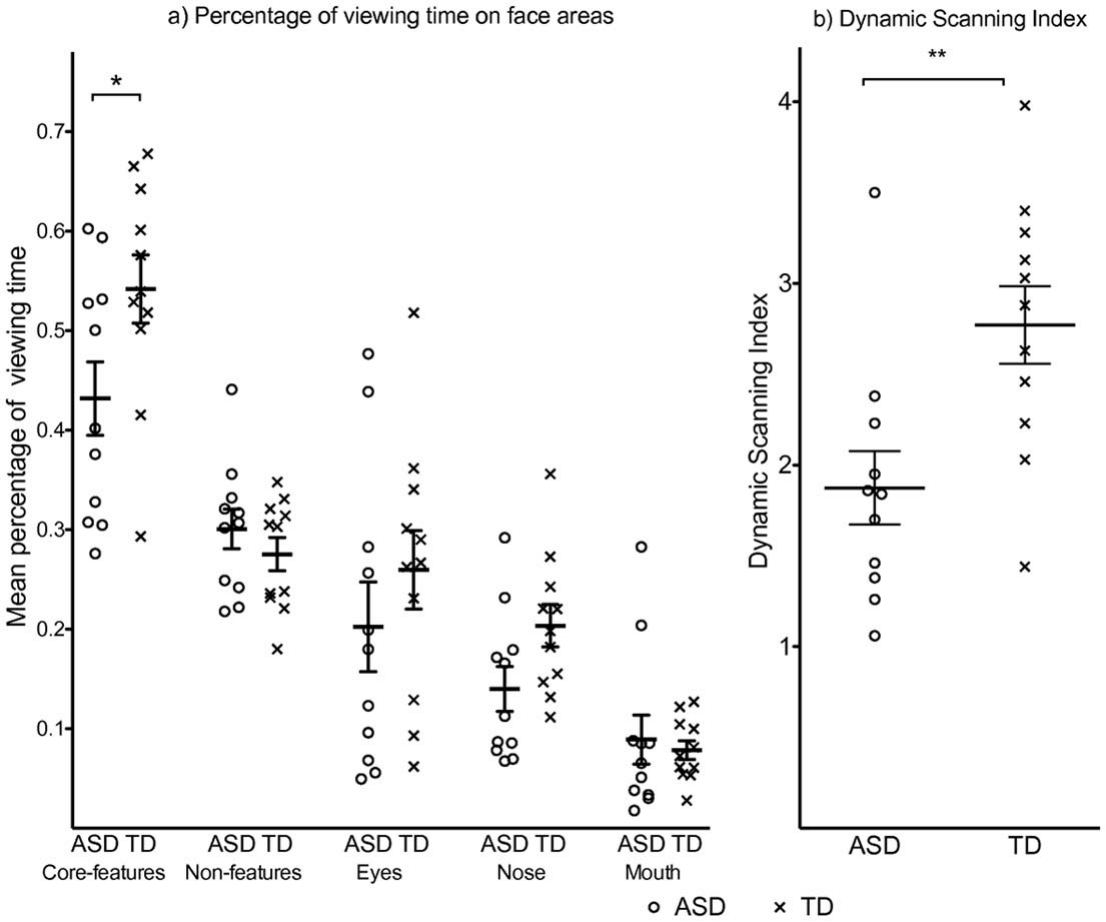
Scatter plots of a) Viewing times on Core-features, Non-core features, Eyes, Nose and Mouth; b) Dynamic Scanning Index, (Horizontal lines indicate group means and standard error means). Difference in group scores: *p<0.05; **p<0.01.

**Figure 4:**
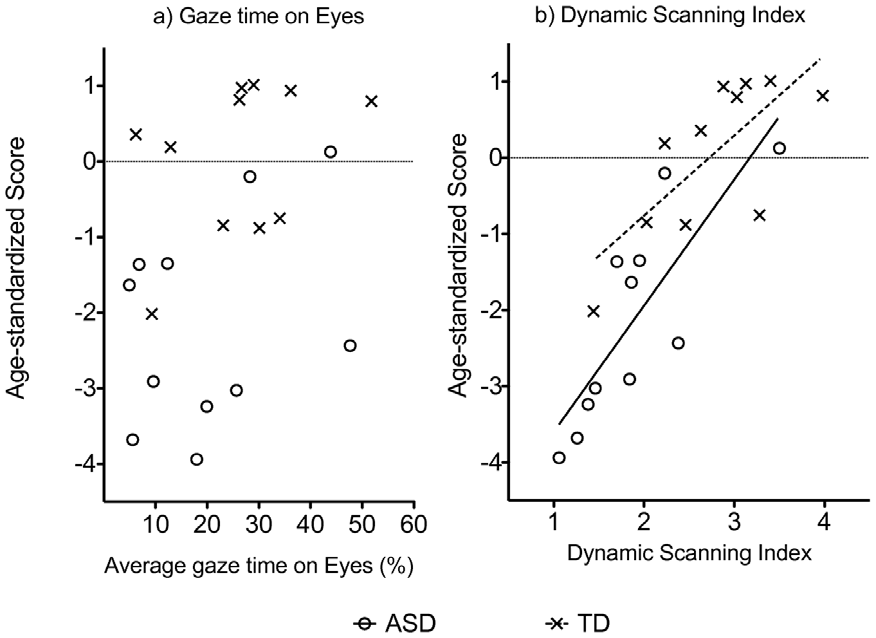
Association between age-standardized face recognition scores and, a) Percentage of gaze time on Eyes (non-significant correlations); b) Dynamic Scanning Index. (significant correlations: ASD group, R^2^ = 0.68; TD group, R^2^ = 0.54).

As shown in Figure 3a, ASD participants and TD controls spent a similar proportion of time looking at the Eyes, (ASD: mean  = 20.25% (SD  = 14.88), TD: mean  = 25.97% (SD  = 13.08), t (22)  = 0.89, *p*  = 0.38). Contrary to predictions, there was no association between looking time on the Eyes and standardized recognition performance in either the ASD group, r (11)  = 0.38, *p*  = 0.25, or the TD group, r (11)  = 0.33, *p*  = 0.32, (Figure 4a).

Further exploratory analyses showed no significant group differences between gaze times on nose or mouth (*p*’s >.05). However, when we combined the gaze time for the core features, the ASD group looked significantly less at the sum of core features (ASD: 43.19% of gaze time (SD  = 12.24), TD: 54.18% (SD = 11.34), t (20)  =  −2.19, *p*  = 0.04) but slightly, although not significantly, *more* at the non-feature face areas (ASD: 30.06% (SD  = 6.61), TD: 27.54% (SD  = 5.51), *p*>.3) (Figure 3a). When age-standardized scores on the face recognition task were correlated with percentage of viewing times on the interest areas, the only significant association was with the sum of core features in the ASD group, r (11)  = 0.64, *p*  = 0.03, and this was marginally significant in the TD group, r (11)  = 0.57, *p*  = 0.07. However, when we conducted partial correlations, controlling for the total gaze time on the whole face, both correlations became non-significant (ASD: r (8)  = 0.50, *p*  = 0.14; TD: r (8)  = 0.45, *p*  = 0.19).

#### Dynamic scanning

In the ASD group the mean Dynamic Scanning Index was 1.98 (SD  = 0.77), and in the TD group it was 2.89 (SD  = 0.77). This difference was significant, t (20)  = 2.78, *p*  = 0.01, (Figure 3b). Similar results were obtained if the two eyes were considered as a single feature (i.e., if a saccade from one eye to the other did not increment the run count). We divided the Dynamic Scanning Index for each participant by the total gaze time on the face in order to check that the group difference in Dynamic Scanning Index was not simply due to a difference in overall time spent looking at the face. Analyses revealed that group differences remained significant, t (20)  = 2.41, *p*  = 0.03.

**Figure 5:**
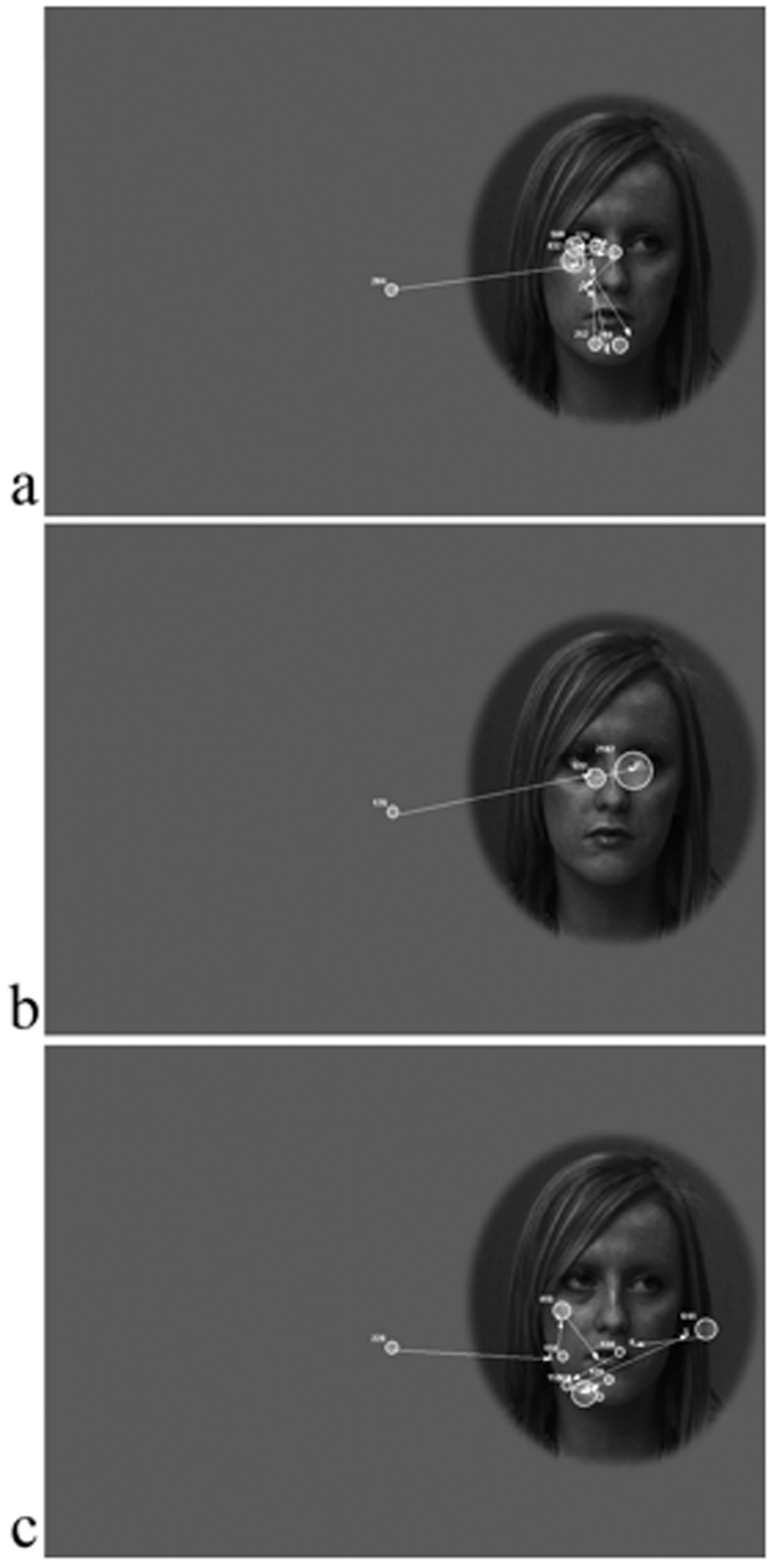
Examples of scanning patterns during face viewing (averted eye gaze condition). (a) TD participant - fixations primarily on core features, with several saccades between features; (b) ASD participant - fixations on eyes, but no saccades between core features; (c) ASD participant - fixations primarily on non-feature face areas.

More importantly, the Dynamic Scanning Index was highly correlated with age-standardized recognition performance in both groups, ASD: r (11)  = .80, *p*  = 0.003; TD: r (11)  = 0.71, *p*  = 0.01 (Figure 4b), consistent with Hypothesis 2. Partial correlations showed that this association remained significant in both groups when controlling for standardized WASI matrices, standardized TROG, and SCQ scores (*p*<.05). Crucially, the correlation remained when average percentage of time spent viewing the face was controlled for, ASD, r (8)  = 0.74, *p*  = 0.02, TD group, r (8)  = 0.62, *p*  = 0.05. Examples of fixation patterns during face viewing are provided in Figure 5.

## Discussion

It has been proposed that atypical face scanning underlies poor recognition of facial identity in ASD [8], [25] but there are few direct tests of this hypothesis. In the current study, eye-movements of ASD and typically developing children were monitored while they learnt pictures of faces. In both groups, the best predictor of performance was the number of times participants’ eye-gaze moved into, and out of, core-feature interest areas (Figure 4b), indicating that successful face recognition is correlated with a pattern of multiple saccades between the core facial features.

We found no significant difference in the amount of time the groups spent looking at the eyes. This contrasts with reports of reduced looking time on the eyes when individuals with ASD watch video clips [31]–[33], although it is consistent with other studies of ASD using static facial images [37]–[39]. However, the key question here was whether individual differences in looking time at the eyes was related to recognition performance, and we found no evidence to support this hypothesis in either ASD or typically developing children (Figure 4a).

The benefit of focusing on *all* the core features and not at the non-features converges with the most compelling result from our data – that greater movement between core features was highly associated with face recognition ability. Subsequent analyses showed that this result was not mediated by indices of general cognitive ability or degree of autistic symptoms. Moreover, although the number of participants in each group was relatively small, the significant association was clearly present in both ASD and typically developing groups, which demonstrates that the effect is replicable across samples. We suggest, therefore, that moving eye-gaze between the core features of a face is a crucial factor in face recognition in ASD and typically developing children.

Charawarska and Shic [25] drew similar conclusions, suggesting that if an ASD child (age 2 or 4 years) focused exclusively on the eyes without distributing attention to all the core-features, their recognition ability would be compromised. However, we note that any causal relationships between movement of eye-gaze, and face recognition ability are currently undetermined. That is, aberrant scanning might lead to poor face recognition, or might be a consequence of an individual’s already poor face recognition skills. Alternatively the relationship may reflect some common factor underlying both reduced eye-movements and poor recognition ability.

Nevertheless, previous studies have suggested moving eye gaze between facial features allows spatial relations to be determined, and that a failure to do this inhibits the formation of a unified visual percept of a face [44]. Such configural or holistic information is thought to be particularly important for accurately discriminating between facial identities [24], [62]–[64] and it is interesting to note that a number of studies have reported reduced holistic processing of faces in ASD. For example, it was found that ASD children were just as good at recognizing facial features presented in isolation as typically developing controls, but were worse than controls at recognizing features presented in the context of whole faces [65], suggesting they were less inclined to make use of the available information of spatial relations [62]. Thus, in our participant sample, the apparent association between a lack of movement and poor face recognition skills might be explained by reduced use of configural/holistic face information.

An interesting comparison here is with individuals with developmental prosopagnosia (DP), a condition in which impaired face recognition occurs in the absence of any acquired brain damage, and in the context of normal low-level visual functioning [66], [67]. In a case study of adults with DP [68], aberrant patterns of face scanning were recorded, with attention being directed away from the internal configuration of core features, and towards peripheral face regions. The authors hypothesized that the disorganized, abnormal scan paths might underlie the impaired face recognition skills that characterize the condition. This hypothesis is largely consistent with results of our own study, however here we are able to demonstrate that it is dynamic saccades between core features that most strongly predict recognition ability, and also to extend the findings to at least two other participant populations. Progress in understanding this relationship will likely be made if experimental results across multiple developmental disorders are considered in combination with individuals developing typically.

The variable face recognition scores within the ASD group demonstrate that facial identity recognition difficulties, like virtually all ASD symptoms, are not present in all individuals on the autistic spectrum therefore its utility as a potential diagnostic marker of the condition is limited. However, poor face recognition skills are clearly evident in many ASD children, thus possible interventions are worth considering. In one study, Schmalzl, et al [22] reported aberrant scan paths in a 4-year-old child initially identified as having DP, but who was later found to meet diagnostic criteria for ASD [69]. An intervention program aimed at directing the child’s attention towards the core features of faces led to significant improvements in recognition of familiar and unfamiliar faces at follow-up assessment one month later [22]. Whilst no comparison data was available, their findings suggest that a failure to attend to core features of the face was indeed a significant factor underlying poor recognition ability in this child.

As noted earlier, our experiment was designed to examine scan paths on faces during a learning phase, however studies such as [22] suggest that scan paths during *recognition* are also important. Furthermore, it is currently unclear how scan paths during encoding and recognition differ from each other, making this is an important question for future research.

### Conclusions

In this study we directly tested hypothesized correlations between visual scan paths of faces, and recognition memory for faces in ASD and in typically developing children. Our analyses revealed that superior recognition performance was strongly associated with the degree of eye-movement between the core features of a face during encoding. Future research would be well placed in confirming the causal directionality of this association, which may then provide a useful basis for developing intervention techniques to effectively improve face recognition ability in ASD and other populations.
